# Laser assisted pulmonary metastasectomy promises a low local recurrence rate

**DOI:** 10.1038/s41598-024-56566-5

**Published:** 2024-03-12

**Authors:** Ahmad Shalabi, Ahmed Ehab, Sundus F. Shalabi, Gudrun Kugler, H.-J. Schäfers, Thomas Graeter

**Affiliations:** 1 Thoracic and Vascular Surgery Department, SLK Lung Medical Center Löwenstein, Löwenstein, Germany; 2 Pulmonary Medicine Department, SLK Lung Medical Center Löwenstein, Löwenstein, Germany; 3 Pulmonary Medicine Department, Mansoura University, Mansoura, Egypt; 4 Faculty of Medicine, Arab American University, Jenin, Palestine; 5 Cardiovascular and Thoracic Surgery Department, Saarland University Medical Center, Homburg/Saar, Germany

**Keywords:** Cancer, Risk factors

## Abstract

Pulmonary metastasectomy (PM) is consensually performed in a parenchyma-sparing manner to preserve functionally healthy lung tissue. However, this may increase the risk of local recurrence at the surgical margin. Laser assisted pulmonary metastasectomy (LPM) is a relatively recent innovation that is especially useful to resect multiple metastatic pulmonary nodules. In this study we investigated the rate of local recurrence after LPM and evaluated the influence of various clinical and pathological factors on local recurrence. Retrospectively, a total of 280 metastatic nodules with different histopathological entities were studied LPM from 2010 till 2018. All nodules were resected via diode-pumped neodymium: yttrium–aluminum-garnet (Nd:YAG) 1,318 nm laser maintaining a safety margin of 5 mm. Patients included were observed on average for 44 ± 17 months postoperatively. Local recurrence at the surgical margin following LPM was found in 9 nodules out of 280 nodules (3.21%). Local recurrence at the surgical margin occurred after 20 ± 8.5 months post operation. Incomplete resection (*p* =  < 0.01) and size of the nodule (*p* = < 0.01) were associated with significantly increased risk of local recurrence at the surgical margin. Histology of the primary disease showed no impact on local recurrence. Three and five-year survival rates were 84% and 49% respectively. Following LPM, the rate of local recurrence is low. This is influenced by the size of the metastatic nodules and completeness of the resection. Obtaining a safety margin of 5 mm seems to be sufficient, larger nodules require larger safety margins.

## Introduction

The lung is one of the most common organs to have metastases from different primary malignancies. Accordingly, pulmonary metastasectomy (PM) is the second most performed operation in thoracic surgery^1^.

Per definition, and in terms of tumor, node, metastasis (TNM) staging, presence of distant metastases reflects an advanced stage of the disease requiring systemic therapy. This represents most of cancer patients. However, it could be furthermore subclassified into wide-spread metastatic disease and oligometastic disease which is defined by the presence of 1 – 5 metastases^2^. Well selected patients with oligometastases would undergo PM with a curative intent and could have a 5-year survival reaching up to 64%^3^. This special entity for oligometastases justifies the widespread of PM as a therapeutic option for these selected patients^4^. While there is an abounded literature on primary lung cancer, evidence regarding a safe resection margin in PM is still lacking or unclear^5^. PM is an art of balance between achieving complete resection (R0 resection^6^) and preservation of lung parenchyma which directly corelates with preservation of pulmonary function^1,7^. As most of metastatic lung nodules are located peripherally within the lung, they are usually resected as a wedge or enucleated irrespectively to segmental anatomy of the lung. Using staplers is widely considered the standard technique for PM^1^. Larg or more centrally located nodules require anatomical resection in means of lobectomy or rarely pneumonectomy.

Complete resection of metastases is linked with better outcomes^7–9^. The various advantages of parenchyma-sparing PM were downsized by the occurrence of local recurrence at the surgical margin^3,10,11^. Local recurrence in its core is nothing but a form of incomplete resection at a microscopic level. This entity has not been sufficiently addressed, perhaps only recently more detailed analysis has been brought up in concern of pulmonary metastases from colorectal origin^8,10,12^. In general, local recurrence at the surgical margin after PM is estimated to occur in 9—31% of PM^8,13,14^. Local recurrence at the surgical margin has molecular-biological reflections along the surgical properties; tumour biology, nodule size, the amount of undetected micro metastases (floating cancer cell clusters (ASFC)) and, most importantly, the completeness of resection are proven factors of significance^8,11,14^. However, this important histo-biological information is only available postoperatively after pathological examination^10^.

Nd:YAG laser is a relatively recent innovation in PM that was introduced to promote parenchyma-sparing PM and to enable the resection (enucleation) of a higher number of nodules or eventually repetitive resections^1,10,15^. LPM causes vaporization and coagulation at the resection site forming a 5 mm coagulation rim^13,16,17^. Here, we assumed that elevated temperature in the coagulation zone would destroy possible recessive tumor cells and thereby increasing safety margin around the resected nodules. The evidence for this in the literature is scarce. The purpose of this study is to estimate the rate of local recurrence at the surgical margin after LPM and evaluate the influence of various clinical and pathological factors on the occurrence of local recurrence following LPM.

## Patients and methods

### Patients

Patients cohort was identified using intraoperative coding and documentation. Patients data who underwent operations with the code ‘laser-assisted pulmonary metastasectomy’ were collected. Between 2010 and 2018, 220 patients with different primary malignant diseases underwent LPM at the department of thoracic surgery at Löwenstein lung medical center, Löwenstein, Germany. A retrospective analysis based on medical and radiological records was performed. This study was approved by the ethical committee of the state medical association of Baden-Württemberg (F-2020–014). Hence, methods were performed in accordance with the relevant guidelines and regulations. Written informed consent regarding the operative intervention and the pathological examination and analysis of the obtained samples was obtained from each patient.

### Study design, inclusion & exclusion criteria

**Figure 1:**
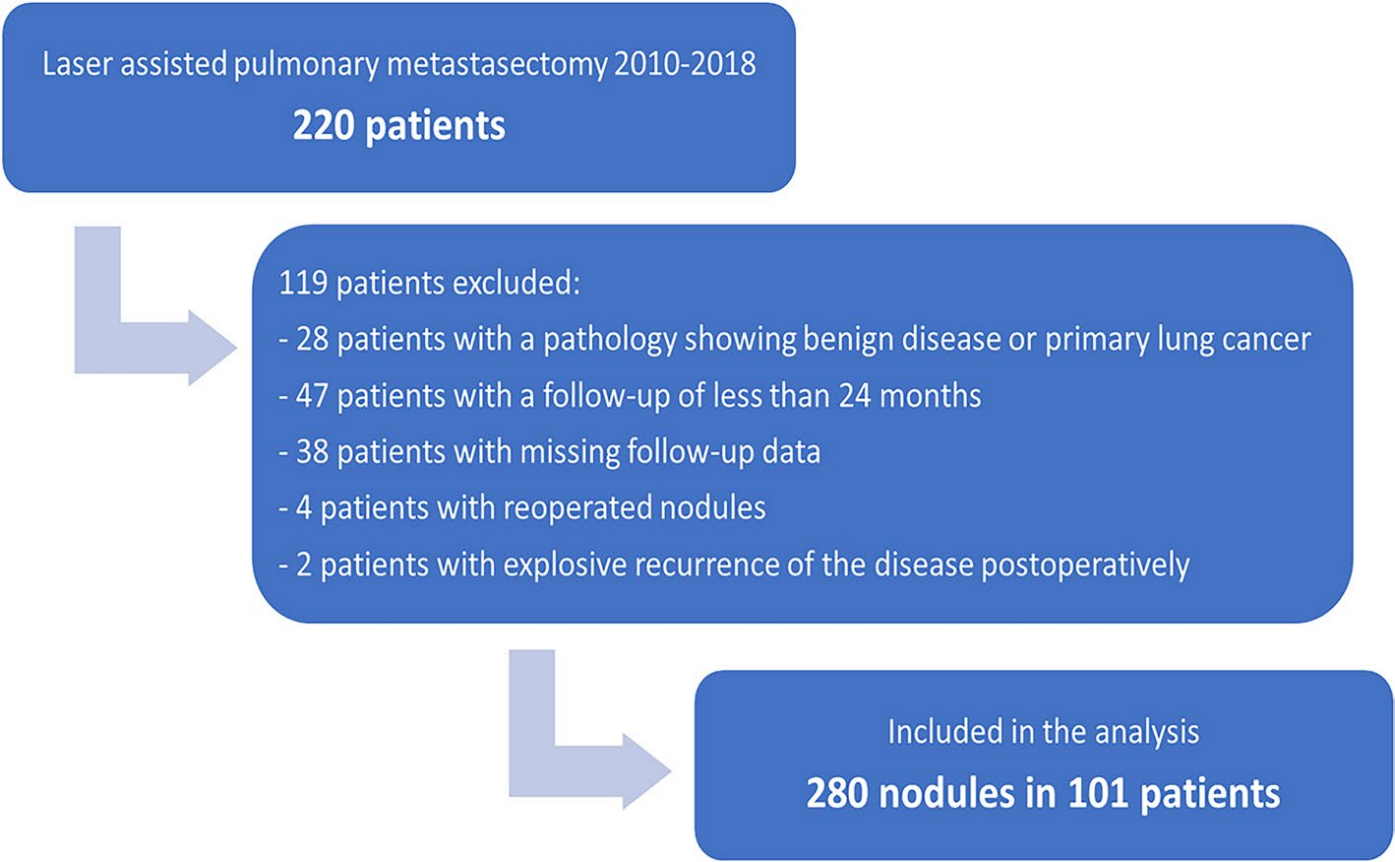
Inclusion flowchart showing the criteria for exclusion.

The main investigated outcome was the local recurrence rate at the surgical margin after LPM. Secondary outcomes were to identify risk factors that influence local recurrence at the surgical margin and overall survival (OS). Patients were followed up and the risk for local recurrence at the surgical margin per nodule resected was analysed. Follow-up was performed with computed tomography (CT) of the chest performed by the surgical team in the outpatient clinic or by the patient’s oncologist or pulmonologist. Postoperative follow-up imaging was then reviewed for evidence of local recurrence at the surgical margin. Follow-up CTs were reviewed by a board-certified radiologist or by the surgeon himself. Exclusion criteria was: macroscopic incomplete resection (R2^6^), reoperated nodules after local recurrence, aggressively progressive disease recurrence hindering the follow-up of the resected nodules, no follow-up CT/data or a follow-up time of less than 24 months (Fig. 1). In cases where intraoperative diversion from plan was necessary, conversion to segmentectomy for example, these nodules, resected without using Nd:YAG laser, were excluded from the study but not the whole patient.

Postoperative follow up is performed at our outpatient clinic or by the referring physician; usually an oncologist or specific field specialist, such as a dermatologist for melanoma. Our local scheme consists of follow up CTs at 3, 6, 12, 18 and 24 months, and annually afterwards.

### Data collection & variables definitions

Data collection for patients followed up in our outpatient clinic was performed by reviewing their local medical records. Data collection for patients followed up by their oncologist or pulmonologist required getting access to their latest records. Oncologists and pulmonologists were contacted and asked to send copies of these records. Disease-free interval (DFI) was defined as the interval between the resection of the primary malignancy and the first detection of pulmonary metastases. Nodule size was defined as the size measured on gross pathological examination. Completeness of the resection (R status) was given by the pathologist; when tumor cells reached the coagulation zone, R status was assigned as ‘‘Rx’’. Local recurrence at the surgical margin was defined as postoperative tumor development at resection line detected on CT by the radiologist or by the surgical team, or pathologically confirmed after resection.

### Surgical technique

Limax®, a diode-pumped Nd:YAG laser (KLS martin, Germany) was used. The whole lung was to be bimanually palpated by the surgeon and all nodules identified. Starting from the nearest pleural surface, nodules were enucleated using the laser beam applying a 5 mm safety margin. This margin was optically estimated and assured by the surgeon for each nodule. The tumor-bed would be coagulated for a second time after nodule resection. Large or deeply parenchymal defects after resection were sewed, small and peripheral defects were only coagulated.

Operative approach (VATS vs thoracotomy) was determined by the surgeon considering the location and number of the nodules and the patient’s functional status. In case of bilateral disease, staged resections were performed 6 weeks apart. All LPM were performed under general anaesthesia with single lung ventilation in lateral decubitus position. Clinical completeness of the resection was fulfilled for each nodule.

### Statistical analysis

Cox proportional hazard model was used to investigate the factors associated with increased risk of local recurrence at the surgical margin after LPM in univariate and multivariate analyses. All p values reported are of two-sided tests, and the significance level was set to less than 0.05. Statistical significance of each factor's effect on local recurrence was assessed using the logrank test. All analyses and plots were performed using the ‘survival’ and ‘survminer’ packages in R® version 4.0.3.

## Results

### Patient characteristics

A total of 280 nodules were studied and analysed among the 101 patients included in this study. On average, each patient had 2.77 nodules while 41 patients had single nodule disease. Among the cohort, 65 patients (64.3%) were males with a mean age of 58.8, and 36 patients (35.6%) were females with a mean age of 66.5. In general, the mean age at the time of operation was 65.7 years (range: 27 – 82 years ± 10.8). Baseline data per patient and per nodule are described in Tables 1 and 2. A total of 129 operations were performed, 5 of them per VATS. Patients included were observed on average for 44 ± 17 months post operatively.

**Table 1:** Baseline data per patient.

	Number (percent)	Local recurrence/patient (percent)
Sex	n = 101	
Male	65 (64.36%)	5 (5/65 = 7.6%)
Female	36 (35.64%)	2 (2/36 = 5.5%)
Spread of the disease	n = 92	
Bilateral disease	54 (58.6%)	5 (5/54 = 9.2%)
Ipsilateral disease	38 (41.3%)	2 (2/38 = 5.2%)
Single nodule	41 (40.6%)	2 (2/41 = 4.8%)
Multiple nodules	60 (59.4%)	5 (5/60 = 8.3%)
DFI	n = 92	
DFI < 36 m	61 (66.3%)	4 (4/61 = 6.5%)
DFI ≥ 36 m	31 (33.7%)	2 (2/31 = 6.4%)
Perioperative chemotherapy	n = 90	
Chemotherapy	67 (74.4%)	6 (6/67 = 8.9%)
No chemotherapy	23 (25.5%)	1 (1/23 = 4.3%)
OP approach	n = 101	
Anterolateral-thoracotomy	96 (95.04%)	7 (7/96 = 7.29%)
VATS	5 (4.95%)	0
Lymphnode metastases	n = 101	
Present	7 (6.93%)	0
Abscent	94 (93.06%)	7 (7/94 = 7.44%)
Total	101	7 (7/101 = 6.93%)

DFI, Disease free interval.

**Table 2:** Baseline data per nodule.

	Number n = 280 (percent)	Local recurrence/nodule (percent)
Sex		
Male	183 (65.36%)	7 (7/183 = 3.83%)
Female	97 (34.64%)	2 (2/97 = 2.06%)
R status		
R0	217 (77.50%)	5 (5/217 = 2.30%)
Rx	63 (22.50%)	4 (4/63 = 6.34%)
Size		
< 12 mm	188 (67.14%)	3 (3/188 = 1.59%)
≥ 12 mm	92 (32.85%)	6 (6/92 = 6.52%)
Spread of the disease	n = 268	
Bilateral disease	203 (75.74%)	7 (7/203 = 3.44%)
Ipsilateral disease	65 (24.25%)	2 (2/65 = 3.07%)
Location		
UL	108 (38.57%)	4 (4/108 = 3.70%)
ML	31 (11.07%)	1 (1/31 = 3.22%)
LL	141 (50.35%)	4 (4/141 = 2.83%)
Total	280	9 (9/280 = 3.21%)

UL, Upper lobe; ML, middle lobe; LL, lower lobe.

The most common primary malignancy was colorectal carcinoma (48.5%) followed by renal cell carcinoma (21.7%). Metastases from primary lung cancer contributed to 8.9% of the patients, while other types of cancer formed the rest with 20.8%. Perioperative chemotherapy (adjuvant therapy for the primary disease or as adjuvant therapy after PM) for was administered in 67 (74.4%) patients, this varied according to the primary disease and its stage. Clinical completeness of the resection was achieved in all patients and for each nodule. Rx, determined by the pathologist when the tumor cells reached the coagulation zone, was found in 63 (22.5%) nodules. Lymphadenectomy was performed in 92 patients (91.0%). Of whom, 7 patients (7.6%) showed evidence of lymph node metastases. Postoperative complications were seen in 10.8% of patients after LPM; these were mainly postoperative infection *(empyema, pneumonia, *etc.*…)* and prolonged air leak.

### Local recurrence and associated factors

After a minimum follow-up of 24 months postoperatively, local recurrence at the surgical margin was detected in 9 nodules (3.2%) in 7 patients (6.9%). Local recurrence occurred on average after 19.6 ± 8.6 months postoperatively. 7 out of 9 nodules (77%) with local recurrence were pulmonary metastases from colorectal origin. Local recurrence was found in 5% of colorectal metastatic nodules. All 9 nodules were reoperated, local recurrence at the surgical margin were resected. Local recurrence was influenced significantly by the size of the nodule (hazard ratio (HR): 1.08, 95% CI: 1.0%-1.1%, *p* = < 0.001). Size was significantly higher in nodules with local recurrence (*p* = 0.02, *Welch Two Sample t-test*) Nodules with local recurrence had an average size of 17.3 ± 8.3 mm (median 17) while nodules which didn’t develop local recurrence had an average size of 9.5 ± 6.5 mm (median 8). Moreover, HR of the local recurrence showed a significant size cut-off point of 12 mm in largest diameter (HR: 4.08, 95% CI: 1.2%-16.3%, *p* = 0.03). Location of the nodule according to the anatomical lobes of the lung didn’t affect local recurrence (*p* = 0.97).

**Figure 2:**
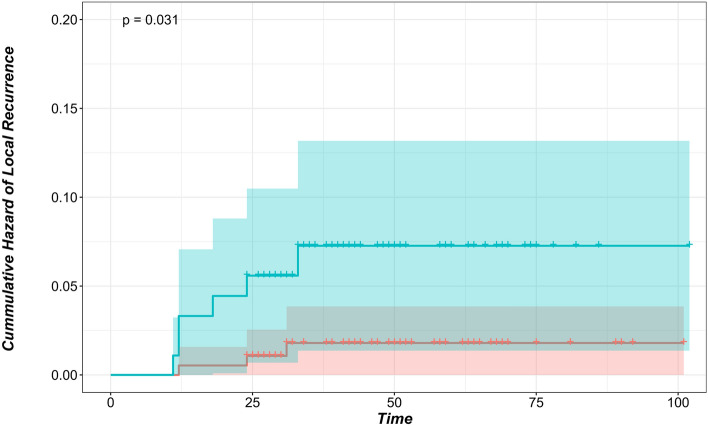
Cumulative hazard of local recurrence for the size of the nodule with 12 mm as a cut-off point. Time in months, green curve: nodules ≥ 12 mm, red curve: nodules < 12 mm. *p* = 0.03 (logrank test).

Completeness of resection (R status) had a significant impact on local recurrence (HR: 12.2, 95% CI: 2.5%-58.7%, p < 0.001). 6.3% of nodules with Rx status had local recurrence after LPM compared to 2.3% of nodules with R0 status. Cumulative hazard for local recurrence according to R status is demonstrated in (Fig. 2). Assessing the factors for risk of local recurrence per patient as well as per nodule was in both univariate and multivariate analysis. None of the other investigated factors seemed to have significant influence on local recurrence. A trend was observed in patients with bilateral disease, but this was statistically insignificant. The effect of primary disease histology on local recurrence could not be seen in this small cohort with colorectal dominance.

### Survival analysis

Survival related data could be collected from 96 (95%) patients; 21 (21.8%) patients died, 75 (78.1%) were then censored. Three and five-year OS rates were 84% and 49% respectively for all patients. Median survival for patients who had local recurrence was 45 months whereas patient's who did not develop local recurrence had a median survival of 49 months, this difference was not significant. HR: 0.84, 95% CI: 0.35%-2.02%, *p* = 0.7).

## Discussion

Adopting a lung parenchyma-sparing PM technique is critically important considering the possible need for further future resections; bilateral disease or pulmonary recurrence. Occurrence of new metastatic nodules after PM or recurrence at a previous resection site is not uncommon. This can be as high as 75% in certain populations, such as patients with colorectal cancer or soft tissue sarcoma. Functionally fit patients would appeal here a second or a third resection^18^. LPM is introduced to serve this concept; for a given metastatic lung nodule of 25 mm, LPM resulted in a 7-times reduction in lung volume lost compared to standard wedge resection with stapler^19^. Here, Nd:YAG- laser presents itself capable to resect a higher significant number of pulmonary metastases and reduce the need for lobectomy without negative influence on survival^1,15,20–22^.

**Figure 3:**
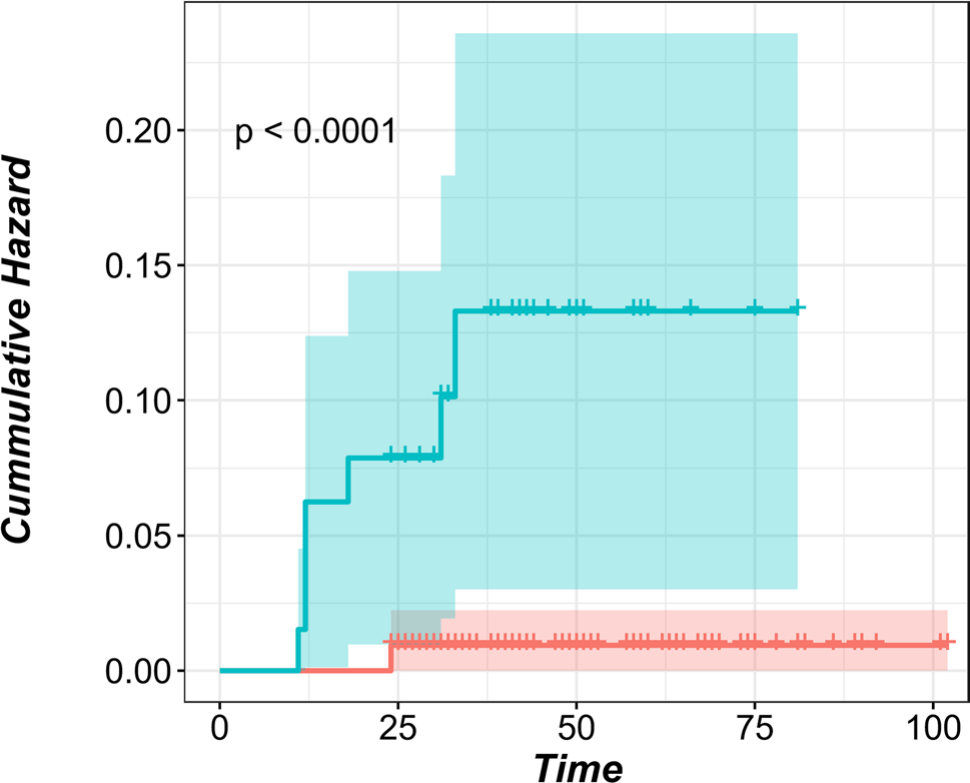
Cumulative hazard of local recurrence for R status. Time in months, green curve: Rx nodules, red curve: R0 nodules. *p* < 0.001 (logrank test).

Our study included 280 metastatic pulmonary nodules with different histologies from 101 patients who underwent a total of 129 LPMs. After a minimum postoperative follow-up of 24 months, 9 nodules (3.2%) from 7 patients (6.9%) showed local recurrence at the surgical margin after an average of 19.6 ± 8.6 months after the operation date. Of the 9 nodules with local recurrence, 7 were pulmonary metastases from colorectal origin. Nevertheless, the effect of primary disease histology on local recurrence could not be seen in these 101 patients dominated by colorectal disease as the results of both the univariate and multivariate analysis were insignificant. In each patient, a safety margin of approximately 5 mm was maintained for each resection to ensure the clinical completeness of the resection for each nodule. Completeness of resection (R0) is considered the key factor for avoiding local recurrence^23,24^. After LPM, it is difficult and sometimes impossible to histopathologically confirm a complete resection due to the vaporization of the resection line and the pronounced coagulation zone. If tumour cells reach the coagulation zone at the edge of the specimen, the pathologist would call it Rx ‘undetermined’, even though another 5–10 mm of vaporization and coagulation zone is yet to follow. Therefore, the completeness of the resection should be clinically evaluated^15,24,25^. Rx was assigned by the pathologist for 63 (22.5%) nodules. Four nodules (6.34%) showed local recurrence of all Rx nodules compared to only 2.3% local recurrence for R0 nodules (*p* < 0.001) (Fig. 3). This suggests that despite the coagulation zone on both sides of the resection line, some of the Rx resections were incomplete (R1). Even when complete resection was histopathologically confirmed (i.e. R0), local recurrence occurred. Despite being partially understood, this phenomenon is known and observed in non-laser PM. Satellite micrometastases due to aerogenous spread, which is pathognomonic for pulmonary malignancies, have been presented to explain this issue^8,11,13^. ASFC is seen predominantly in metastases of colorectal origin^13^. Kawaguchi et al. showed recently that mucus extension in resected mucinous tumour hosts had an increased risk for local recurrence after PM despite R0 resection^26^. These factors persist as risk factors for local recurrence in R0 resections in LPM.

Local recurrence rate at the surgical margin after non-laser PM is relatively high, with published reports ranging from 9 to 30%^8,12,24,27^. A 3.2% local recurrence rate after LPM manifests the protective effect of the coagulation zone produced in LPM, especially if ASFC is suspected. Also, of note, almost all studies investigating local recurrence after non-laser PM addressed colorectal pulmonary metastases. In our study, the population with colorectal cancer had a 5.0% local recurrence rate, which is still less than non-laser PM. Shiono et al. tried to explain this issue: as both stapler jaws close in wedge resections, the surgical margin could be compromised^8^. Moreover, the surgical margin could be more compromised in a VATS-based wedge resection^15^.

In their retrospective comparison of surgical outcomes after LPM and non-laser PM, Franzke et al. commented on local recurrence as a secondary outcome^15^. They reported an interestingly low local recurrence rate of 0.8% after LPM vs 3.1% after non-laser PM. However, around 60% of their patients in both groups were followed up for less than 24 months, enhancing the possibility of latterly undetected local recurrence. Moreover, detection of local recurrence after LPM in the early postoperative phase could be quite difficult due to postoperative residual changes. Minimal local tissue damage or haematoma after LPM demonstrates the same radiological appearance of local recurrence early on.

Local recurrence significantly correlates with nodule size (*p* = 0.02). Nodules with local recurrence had an average size of 17.3 ± 8.3 mm (median 17), while nodules that did not develop local recurrence had an average size of 9.5 ± 6.5 mm (median 8). This is in accordance with published studies that investigated local recurrence after non-laser PM^13,28^. A significant cut-off point was found at 12 mm (*p* = 0.03), where tumours larger than 12 mm had an increased risk of local recurrence (Fig. 2). Nelson et al. reported a 12% risk for 1–2 cm tumours and an increased risk (31%) in tumours larger than 2 cm^28^. Welter and colleagues successfully demonstrated in two different publications supported by histopathological analyses that an increase in tumour size reflects an aggressive local growth and an increase in the number of satellite cells and their distance from the tumour^11,13^. Consequently, an increased safety margin for larger tumours was recommended. Affirmatively, Nelson et al. demonstrated the increased risk for local recurrence in larger tumours diminished when a sufficient margin applied^28^.

The median survival for patients with and without local recurrence was 45 and 49 months, respectively. No significant difference in OS was found in respect to local recurrence, this is probably because all patients with local recurrences were reoperated (i.e. recurrence was resected). Jaklitsch et al. confirmed this assumption reporting a > 33% 5-year Survival in up to 5 operative sessions after sequential PM in patients with various primary tumours^29^. Two-year survival dropped to 19% as local control was lost.

VATS LPM was performed in only five patients. None of these patients developed local recurrence. Although these patients had fewer and more peripherally located and smaller nodules, this presents VATS LPM as a doable and reliable option. However, evidence in support of VATS LPM is currently lacking, although positive experiences with this technique are emerging^30^.

### Limitations

This study contained some limitations, so caution is required when interpreting the results. Nevertheless, these limitations should not disrupt the main finding that the local recurrence rate at the surgical margin after LPM is low. This study was retrospective and included a relatively small number of patients and nodules. The 129 LPMs in this study were performed by different surgeons, and the specimens were reviewed by different pathologists, thus raising the margin of human error. The high number of censored patients and the low number of patients with local recurrence weakened the statistical analysis regarding survival and masked the factors associated with increased risk for local recurrence at the surgical margin. The primary malignancies were heterogeneous and not equally presented in this population with colorectal dominance. This limits the generalizability of the results and conclusions.

### Conclusion

The local recurrence rate at the surgical margin after LPM is low when a 5-mm safety margin is obtained. Larger nodules require larger safety margins.

## Data Availability

The dataset used and analysed during the study is available from the corresponding author on reasonable request.

## References

[1] Panagiotopoulos N, Patrini D, Lawrence D, Scarci M, Mitsos S (2018). Pulmonary metastasectomy and laser-assisted resection. J. Thorac. Dis..

[2] Lievens Y (2020). Defining oligometastatic disease from a radiation oncology perspective: an ESTRO-ASTRO consensus document. Radiother. Oncol..

[3] Shiono S (2005). Histopathologic prognostic factors in resected colorectal lung metastases. Ann. Thorac. Surg..

[4] Lussier YA (2012). Oligo-and polymetastatic progression in lung metastasis (es) patients is associated with specific microRNAs. PLoS One.

[5] Sawabata N (2004). Optimal distance of malignant negative margin in excision of nonsmall cell lung cancer: A multicenter prospective study. Ann. Thorac. Surg..

[6] Hermanek P, Wittekind C (1994). The pathologist and the residual tumor (R) classification. Pathol. Pract..

[7] Handy JR (2019). Expert consensus document on pulmonary metastasectomy. Ann. Thorac. Surg..

[8] Shiono S (2005). Predictive factors for local recurrence of resected colorectal lung metastases. Ann. Thorac. Surg..

[9] Pastorino U (1997). Long-term results of lung metastasectomy: Prognostic analyses based on 5206 cases. J. Thorac. Cardiovasc. Surg..

[10] Higashiyama M, Tokunaga T, Nakagiri T, Ishida D, Kuno H, Okami J (2015). Pulmonary metastasectomy: outcomes and issues according to the type of surgical resection. Gen. Thorac. Cardiovasc. Surg..

[11] Welter S, Theegarten D, Trarbach T, Maletzki F, Stamatis G, Tötsch M (2011). Safety distance in the resection of colorectal lung metastases: A prospective evaluation of satellite tumor cells with immunohistochemistry. J. Thorac. Cardiovasc. Surg..

[12] Higashiyama M (2002). Intraoperative lavage cytologic analysis of surgical margins as a predictor of local recurrence in pulmonary metastasectomy. Arch. Surg..

[13] Welter S (2017). Growth patterns of pulmonary metastases: Should we adjust resection techniques to primary histology and size?. Eur. J. Cardio-Thoracic Surg..

[14] Kim S (2011). Pulmonary resection of metastatic sarcoma: prognostic factors associated with improved outcomes. Ann. Thorac. Surg..

[15] Franzke K (2017). Pulmonary metastasectomy – A retrospective comparison of surgical outcomes after laser-assisted and conventional resection. Eur. J. Surg. Oncol..

[16] Kirschbaum A, Palade E, Kayser G, Passlick B (2012). Local effects of high-powered neodymium-doped yttrium aluminium garnet laser systems on the pulmonary parenchyma: An experimental study on the isolated perfused pig lung lobe. Interact. Cardiovasc. Thorac. Surg..

[17] Rolle A, Pereszlenyi A, Koch R, Bis B, Baier B (2006). Laser resection technique and results of multiple lung metastasectomies using a new 1,318 nm Nd:YAG laser system. Lasers Surg. Med..

[18] Welter S, Jacobs J, Krbek T, Poettgen C, Stamatis G (2007). Prognostic impact of lymph node involvement in pulmonary metastases from colorectal cancer. Eur. J. Cardio-Thoracic Surg..

[19] Rolle A, Kozłowski M (2005). Laser resection of lung parenchyma–a new technical and clinical approach. Rocz. Akad. Med. Bialymst..

[20] Rolle A, Pereszlenyi A, Koch R, Richard M, Baier B (2006). Is surgery for multiple lung metastases reasonable? A total of 328 consecutive patients with multiple-laser metastasectomies with a new 1318-nm Nd:YAG laser. J. Thorac. Cardiovasc. Surg..

[21] A. Rolle, A. Pereszlenyi, Laser resection of lung metastasis. *Multimed. Man. Cardiothorac. Surg. MMCTS*, 2005(628), 2004, 2005. 10.1510/mmcts.2004.000570 24414728

[22] Osei-Agyemang T, Palade E, Haderthauer J, Ploenes T, Yaneva V, Passlick B (2013). Pulmonary metastasectomy: an analysis of technical and oncological outcomes in 301 patients with a focus on laser resection. Zentralbl. Chir..

[23] Shiono S (2017). Outcomes of segmentectomy and wedge resection for pulmonary metastases from colorectal cancer. Eur. J. Cardio-Thoracic Surg..

[24] Chung JH (2019). Impact of resection margin length and tumor depth on the local recurrence after thoracoscopic pulmonary wedge resection of a single colorectal metastasis. J. Thorac. Dis..

[25] Macherey S, Doerr F, Wahlers T, Hekmat K (2017). Role of laser resection in pulmonary metastasectomy. Pneumologie.

[26] Y. Kawaguchi *et al.*, Locoregional recurrence via mucus- mediated extension following lung resection for mucinous tumors, pp. 1–10, 2021. 10.1186/s12885-021-08231-7 PMC8080361 33906634

[27] Okumura S (1996). Pulmonary resection for metastatic colorectal cancer: experiences with 159 patients. J. Thorac. Cardiovasc. Surg..

[28] Nelson DB (2019). Surgical margins and risk of local recurrence after wedge resection of colorectal pulmonary metastases. J. Thorac. Cardiovasc. Surg..

[29] Jaklitsch MT (2001). Sequential thoracic metastasectomy prolongs survival by re-establishing local control within the chest. J. Thorac. Cardiovasc. Surg..

[30] Ojanguren A, Karenovics W, Dackam S, Demarchi M, Triponez F (2019). Laser pulmonary metastasectomy by video-assisted thoracic surgery. J. Vis. Surg..

